# Predictors of Positive Video Capsule Endoscopy Findings for Chronic Unexplained Abdominal Pain: Single-Center Retrospective Study and Meta-Analysis

**DOI:** 10.3390/diagnostics11112123

**Published:** 2021-11-16

**Authors:** Wonshik Kim, Beomjae Lee, Ahyoung Yoo, Seunghan Kim, Moonkyung Joo, Jong-Jae Park

**Affiliations:** Department of Gastroenterology, Korea University Guro Hospital, Seoul 08308, Korea; ws907568@gmail.com (W.K.); person88@naver.com (A.Y.); drshkim@korea.ac.kr (S.K.); latyrx@korea.ac.kr (M.J.); gi7pjj@korea.ac.kr (J.-J.P.)

**Keywords:** chronic abdominal pain, video capsule endoscopy, inflammatory markers

## Abstract

Video capsule endoscopy (VCE) is an effective diagnostic modality for detecting small bowel lesions. However, the value of VCE for patients with chronic recurrent abdominal pain (CAP) of unknown etiology remains obscure. We retrospectively analyzed factors that could predict enteropathy based on the medical records of 65 patients with unexplained chronic recurrent abdominal pain (CAP) who were assessed using VCE between 2001 and 2021. We also conducted a systematic review and meta-analysis of the literature to validate our results. The positive findings of 27 (41.5%) of the 65 patients were mostly ulcerative lesions including stricture (*n* = 14, 60.9%) and erosion (*n* = 8, 29.7%). Multivariate analysis identified elevated ESR (OR, 1.06, 95% CI, 1.02–1.1, *p* = 0.004) as a significant risk factor for enteropathy predicted by VCE. Three eligible studies in the meta-analysis included 523 patients with CAP. Elevated C-reactive protein (CRP) (OR, 14.09; 95% CI, 2.81–70.60; *p* = 0.001) and erythrocyte sedimentation rate (ESR) (OR, 14.45; 95% CI, 0.92–227.33; *p* = 0.06) indicated VCE-positive findings in patients with unexplained abdominal pain. Elevated levels of the inflammatory markers ESR and CRP can thus predict positive VCE findings in patients with CAP.

## 1. Introduction

Lesions such as occult gastrointestinal bleeding (OGIB), inflammatory states such as Crohn’s disease, and small bowel tumors are now being screened and diagnosed as a result of advances in video capsule endoscopy (VCE), which is less invasive than enteroscopy [1,2,3].

Conventional modalities cannot always determine the etiology of chronic abdominal pain (CAP). Deficiencies in the diagnosis, treatment, prevention of enteropathy associated with conventional modalities, the aging population, and the prevalence of medication with non-steroidal anti-inflammatory drugs (NSAIDs) and antiplatelet agents have attracted research interest in this regard [4].

The number of patients with inflammatory bowel disease (IBDs), such as Crohn’s disease, is also increasing, particularly in Asia, and the early diagnosis and detection of unexplained abdominal pain caused by small bowel lesions has become an important clinical issue for gastroenterologists [5,6].

VCE is considered a less invasive, more tolerable, and safe option that offers advantages over other diagnostic modalities, but its value to patients with chronic abdominal pain of unknown etiology and the predictive factors associated with enteropathy determined by VCE have remained unclear. Therefore, we aimed to identify risk factors and early predictors to identify the cause(s) of CAP. We also conducted a systemic meta-analysis of the literature. Our results will help to predict early enteropathies such as Crohn’s disease and those induced by drugs and prevent the unnecessary application of VCE; this will decrease health costs as well as the burden on patients and physicians.

## 2. Materials and Methods

### 2.1. Patients

**Figure 1:**
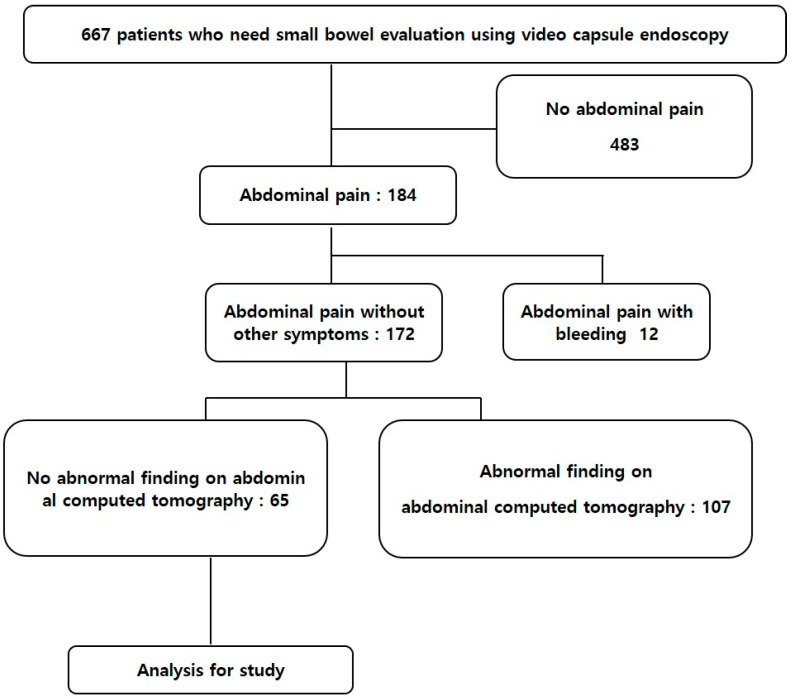
Flowchart showing patient inclusion.

Among the 667 patients who were assessed for overt gastrointestinal bleeding or OGIB, diarrhea, and CAP using VCE at Korea University Guro Hospital between January 2010 and February 2021, data from the capsule registry identified 65 with unexplained CAP who had tested negative in conventional medical examinations, including abdominal–pelvic computed tomography. We analyzed the findings of VCE in these 65 patients as well as their clinicopathological data. Figure 1 shows the flow of patients through this study. The ethics committee at Korea University, Guro Hospital approved this study.

### 2.2. Video Capsule Endoscopy

Patients were assessed by VCE using a MiroCam^®^ (IntroMedic, Seoul, Korea). The bowels were prepared using laxatives, then a video capsule was ingested within 4 h thereafter. The capsule images were recorded through VCE for 12 h, then the receiver was retrieved and the images were analyzed by two expert endoscopists. The IC valve was identified in all patients. Visualization of the mucosa was acceptable for analysis in all patients without serious complications such as capsule retention [7]. The VCE findings were classified as mucosal inflammatory lesions, including erosions, ulceration, and strictures, or tumorous lesions; their associations with abdominal pain were assessed. 

### 2.3. Study Search Strategy

We comprehensively reviewed PubMed, Embase, and the Cochrane Library databases using the search terms, “chronic abdominal pain” “capsule endoscopy,” and “adult” up to 19 August 2021. If data or methodological details were not specified, the first author was contacted by e-mail. The study was excluded if a reply was not received. When studies had investigated more than one group, only the relevant groups were included.

### 2.4. Eligible Criteria and Data Extraction

**Figure 2:**
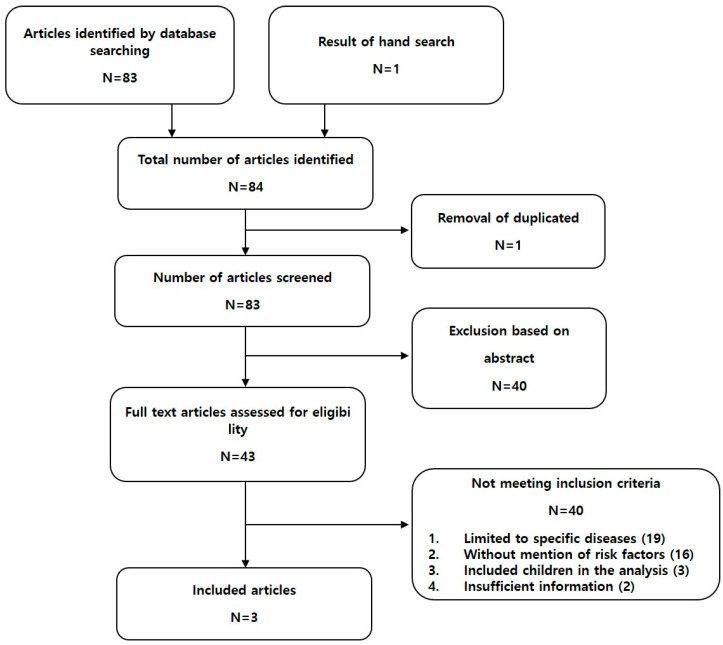
Preferred reporting items for systematic reviews and meta-analyses diagram.

We identified and evaluated 84 titles and abstracts, then further analyzed 43 articles. However, 40 of these did not meet the inclusion criteria. The exclusion criteria comprised studies limited to specific diseases (*n* =19), those without mention of risk factors (*n* = 16), those that included children (*n* = 3), and those with insufficient information (*n* = 2). Finally, three publications were analyzed (Figure 2) [8,9,10].

Supplementary Tables S1 and S2 were summarized of basal characteristics and quality assessment using the QUADAS-2 tool of enrolled studies. Only studies with a case-control design that were published in English, and contained sufficient data to calculate hazard ratios (HR) and 95% confidence intervals (CI) were included in the meta-analysis. If a case-control design was not used, or data were not extractable, the articles were excluded from the aggregated meta-analysis. 

### 2.5. Statistical Analysis

Data were statistically analyzed using Student’s *t*-test and Fisher’s exact test as appropriate. Relationships between significant variables in VCE results and symptoms in patients with CAP were confirmed using univariate and multivariate logistic regression analyses. Heterogeneity of the meta-analysis was evaluated using Q and I^2^. A random effects model was applied when *p* < 0.10, and I^2^ was >50% indicating heterogeneity. Publication bias was visually assessed using Begg funnel plots. Data were statistically analyzed using SPSS software version 20 (IBM Corp., Armonk, NY, USA). Significance was set at *p* < 0.05 for all analyses [11,12,13].

## 3. Results

### 3.1. Baseline Characteristics of Enrolled Patients

The 65 patients comprised 29 men and 36 women. The mean ages of the capsule-negative and -positive groups were 49.42 ± 16.59 (range, 19–84) and 54.11 ± 18.15 (range, 18–79) years, respectively. Table 1 shows the baseline and clinical characteristics.

### 3.2. Findings of Capsule Endoscopy

Findings were positive in 27 of the 65 patients (Table 1), and the diagnostic yield was 41.53%. Twenty-two (57.89%) of 38 patients presented with inflammatory lesions, including mucosal ulcers, erosions, and ulcers with strictures. Of these, seven and six were respectively diagnosed with Crohn’s disease and drug-induced enteropathy. One patient had multiple polyps, another had significant erythema that caused abdominal pain, and 27 (41.54%) were negative. Table 2 summarizes the VCE findings.

### 3.3. Comparison of Clinical and Laboratory Findings between the Capsule Positive and Capsule Negative Groups

Demographic and laboratory findings were compared between the VCE-negative and -positive groups (Table 1). Overall, 27 patients had positive VCE findings (41.53%), and 6 (22.2%) of 27 had been medicated with antiplatelet agents, anticoagulants, and NSAIDs, in the capsule-positive group. However, the two groups did not significantly differ. The activities of inflammatory markers, including ESR and CRP, were significantly higher in the capsule-positive, than negative group (CRP, 19.10 ± 29.94 vs. 1.84 ± 4.33, ESR, 31.25 ± 28.37 vs. 13.54 ±12.35, *p* < 0.01 for both).

### 3.4. Elevated ESR Was a Significant Predictor of Positive VCE

**Figure 3:**
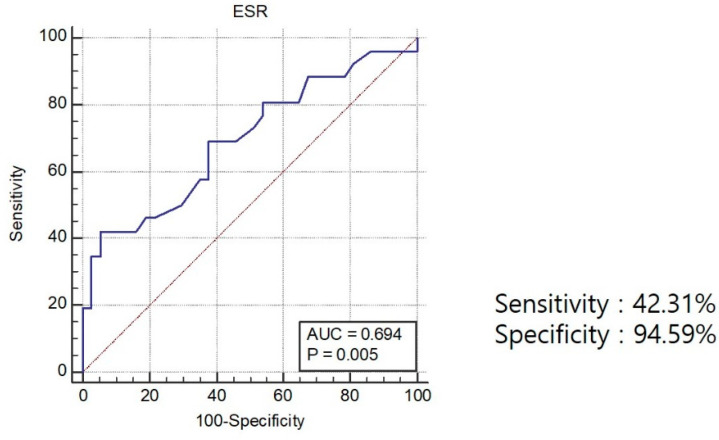
The area under the ROC curve for ESR for predicting a positive finding of VCE in patients with CAP was 0.69 (95% CI 0.57–0.80). ROC, Receiver operating characteristics; CI, confidence interval.

Univariate logistic regression analysis significantly associated elevated ESR (OR 1.05, 95% CI, 1.01–1.08, *p* = 0.006), CRP (OR, 1.12, 95% CI, 1.0–1.24, *p* = 0.042), and platelet counts (OR 1.01, 95% CI 1–1.02, *p* = 0.02) with positive capsule findings. Multivariate logistic regression analysis selected elevated ESR as a significant risk factor for positive VCE findings in CAP (OR, 1.06, 95% CI, 1.02–1.1, *p* = 0.004), (Table 3 and Table 4). By Youden’s J statistic method, we calculated the cutoff value, sensitivity, and specificity of the predicted laboratory parameters for predicting positive VCE findings in patients with CAP. The area under the receiver operating characteristics curve (AUROC) was 0.69 (95% CI, 0.57–0.80). The cutoff for ESR was 31 mm/h, and the sensitivity and specificity were 42.31% and 94.59%, respectively (Figure 3).

### 3.5. Result of Meta-Analysis for Relevant Factors of VCE Positive Findings

Our systematic literature review initially identified 84 articles. However, only three studies that included 523 patients with CAP were finally included in the meta-analysis. Each study identified relevant factors that could predict positive findings of VCE that are thought to be related to symptoms in patients with unexplained chronic abdominal pain.

Shim, K.-N. et al. retrospectively analyzed 110 patients with unexplained chronic abdominal pain. Diagnostic yield was 17.3%. Significant risk factor for positive findings of VCE was weight loss (OR, 18.6; 95% CI: 1.6–222.4, *p* = 0.02) in multivariate analysis [9].

Katsinelos, P. et al. conducted an open-label prospective non-randomized multicenter clinical trial. Diagnostic yield was 44.4%. Elevated ESR and CRP were associated with positive findings of VCE in multivariate regression analysis. [(OR, 67.9, 95% CI: 9.3–310.6, *p* < 0.001) and (OR, 41.5, 95% CI: 6.2–213.4, *p* < 0.001), respectively] [10].

Huang, L. et al. conducted a retrospective study which included 341 patients with CAP. Diagnostic yield: 28.15%. In this study, weight loss (OR, 2.827, 95% CI: 1.938–4.926, *p* = 0.038), hypoalbuminemia (OR, 6.142, 95% CI: 4.129–8.274, *p* = 0.008), elevated ESR (OR, 4.025, 95% CI: 3.178–6.892, *p* = 0.016), and increased CRP (OR, 7.539, 95% CI: 5.365–11.723, *p* = 0.002) were significantly relevant risk factors of the positive finding of VCE [8] (Supplementary Table S3).

**Figure 4:**
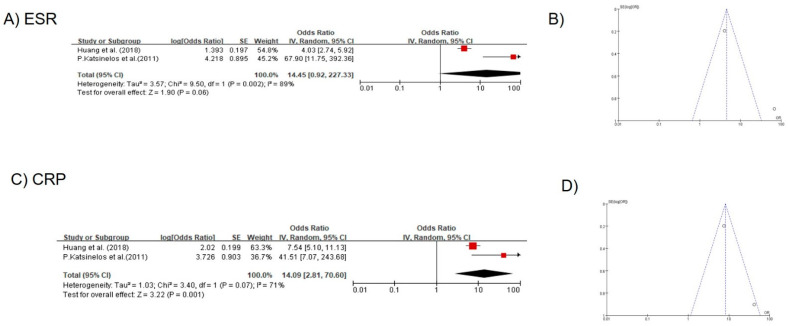
(**A**) forest plot of elevated ESR; (**B**) funnel plots of studies that examined elevated ESR; (**C**) forest plot of elevated CRP; (**D**) funnel plots of studies that examined elevated CRP.

Elevated CRP was identified as a risk factor for positive VCE findings in two of these three studies that involved 413 patients. The VCE findings were abnormal and normal in 128 and 285 patients, respectively. The random effects model was applied due to high heterogeneity between the two studies (χ^2^ = 3.40, *p* = 0.07, I^2^ = 71%). The results of the meta-analysis showed that an elevated CRP value was significant (OR, 14.09, 95% CI, 2.81–70.60, *p* = 0.001), and an elevated ESR was a relevant risk factor for positive VCE findings. The random effects model was applied due to heterogeneity between the two studies (χ^2^ = 9.50, *p* = 0.002, I^2^ = 89%). The results of the meta-analysis showed that the OR of elevated ESR was 14.45 for VCE positivity, but it did not reach statistical significance (95% CI, 0.92–227.33, *p* = 0.06). (Figure 4).

## 4. Discussion

In this study, we showed that elevated ESR was more prevalent in the VCE-positive group than in the VCE-negative group. Univariate and multivariate regression analysis selected elevated ESR as a significant factor for positive VCE findings. Therefore, elevated ESR is an informative laboratory finding for patients with abnormal small bowel lesions. In addition, additional meta-analysis showed that inflammatory markers such as ESR and CRP were significant predictors which could predict positive VCE findings in CAP patients.

VCE has become a powerful non-invasive modality since its introduction to clinical practice in 2001 and since then, there has been a significant advance in the diagnostic ability of VCE in various small bowel lesions [14] It can evaluate whole small-bowel mucosa and can detect abnormalities that were previously undetectable by small-bowel radiography and abdominal pelvic CT, and angiography [15,16,17,18,19,20,21,22,23,24,25].

Obscure gastrointestinal bleeding was the initial main indication for VCE, but this became expanded to include small bowel inflammatory lesions, including Crohn’s disease, celiac diseases, and other inflammatory conditions [6,26,27,28]. A previous systemic review and meta-analysis of suspected Crohn’s disease found a diagnostic OR for VCE of 29 and an AUC of 0.92 [29,30]. However, patients with Crohn’s disease are at risk for capsule retention, and this should be assessed before proceeding with VCE [26,31,32] Besides, due to an extended life expectancy and an increased prevalence of cardiovascular diseases, more patients are medicated with NSAIDs and anti-platelet agents that can damage the gastrointestinal mucosa [33] The prevalence of medication with NSAIDs in healthy populations has also increased the incidence of intestinal injury by 55–75% [34,35,36].

Patients with CAP who do not respond to medical treatment and have no evidence of other organic disease on laboratory, radiologic, and endoscopic examination make gastroenterologists wonder whether the abdominal pain has originated from a functional or an organic cause.

The value and indication of VCE to patients with CAP remains unclear. Previous studies showed that the diagnostic yields of VCE in patients with CAP were reported to vary from 24.2% to 41.9%. The overall diagnostic yield of our study was 41.5%, higher than in previous studies. In our study, 24 cases of mucosal lesions including 23 inflammatory lesions and three cases of tumorous lesions were identified. Among the inflammatory lesions, drug induced enteropathy, six cases (26.1%), Crohn’s disease, seven cases (30.4%), and non-specific, 10 cases (43.5%) were identified.

Since the diagnosis rate is different for each study, it is necessary to clarify the indications for capsule endoscopy in the diagnosis of CAP. Previous studies showed that the diagnostic yield was higher in patients with elevated inflammatory markers, compared with those who had normal laboratory findings [37]. Previous studies have shown that elevated inflammatory markers such as ESR, CRP, and leukocytosis are significant predictors of positive findings in patients with CAP [8,10].

In our study, we showed that elevated ESR was more prevalent in the VCE-positive group than in the VCE-negative group. ESR activity of over 31 mm/h was a significant predictive value of positive VCE findings with patients with CAP (OR, 12.83; sensitivity, 42.31; 95% CI, 23.4–63.1; specificity, 94.59; 95% CI, 81.8–99.3, *p* = 0.002). The high specificity of the ESR cut-off level indicated that if the patients whose ESR level was high should be recommended to undergo VCE for detecting clinically significant small bowel lesions.

Since most previous studies were retrospective studies and enrolled patients were heterogeneous, we performed meta-analysis to verify our results. Systemic meta-analysis showed that inflammatory markers such as ESR and CRP were significant predictors which could predict positive VCE findings in CAP patients. Thus, our results including meta-analysis were in line with those of previous studies [9]. In our retrospective study, mean CRP activity was significantly higher in the VCE positive group, but multivariate analysis could not show the meaningful power to predict a small bowel lesion in patients with CAP. The presumptive reason of this discrepancy could be due to the small number of patients enrolled in our study. A well-designed prospective study with a large number of enrolled patients is needed.

Our study has several limitations. We found that 41.5% of our patients were VCE-positive, which was higher than that of previous studies. This might have been due to selection bias, which is an inherent limitation of retrospective investigations at a single center. Another explanation might be that patients with abdominal pain other than small bowel origin were previously excluded because most patients were referred for evaluations of uncontrolled chronic abdominal pain. Third, only a small number of studies could be included in the meta-analysis. We initially resolved the significant heterogeneity of the analyzed studies by evaluating biological markers using a standardized process. Most VCE studies of patients with CAP have been small-scale and retrospective. Only two of the three selected studies included >100 patients, and their diagnostic yields were 96 (28.15%) of 341 and 19 (17.27%) of 110. Due to the dearth of good-quality prospective studies, we could analyze only very few studies that applied different criteria to measure inflammatory markers such as ESR and CRP. Few studies have assessed the diagnostic predictors of VCE and their effectiveness in CAP; thus, our meta-analysis included only three independent studies of 523 patients, among whom the VCE findings were positive in 147 (overall diagnostic yield: 28.1%). Our literature review revealed only three small, heterogeneous studies that included patients with CAP who had tested negative according to conventional diagnostic modalities. The small number of such patients overall prevented the inclusion of a sufficient number of patients.

Despite the limitations mentioned above, the strength of our study is that we analyzed many patients with CAP, including a meta-analysis, and confirmed that inflammatory markers such as ESR and CRP are important predictors of small intestine lesions in patients with unexplained abdominal pain. Our results can differentiate between patients with CAP caused by lesions of the small intestine and patients with pure functional gastrointestinal disorders. CAP is a common but non-specific symptom, most of which originates from non-organic causes and increases medical costs for evaluation and treatment. Our results suggest a VCE indication of VCE for CAP patients and are expected to reduce the medical costs and gastroenterologist’s efforts through selective performing VCE.

## 5. Conclusions

The inflammatory markers ESR and CRP provided diagnostic performance that was sufficient to predict abnormal VCE findings in patients with CAP that cannot be detected or explained by conventional diagnostic modalities. From our retrospective and meta-analysis study, VCE could be a frontline diagnostic modality to evaluate the unknown CAP patients with elevated inflammatory markers such as ESR and CRP.

## Figures and Tables

**Table 1:** Patients’ Baseline Characteristics.

Variable	Capsule Negative (*n* = 38)	Capsule Positive (*n* = 27)	*p*
Age (year)	49.42 ± 16.57	54.11 ± 18.15	0.28
Gender (M/F)	18/20	11/16	0.06
BMI (kg/m^2^)	22.80 ± 3.75	23.40 ± 3.16	0.54
Smoking	29	23	0.09
Alcohol	26	18	0.81
Medication			0.62
Anti-PLT agent	5	2	
NSAIDs	2	4	
Comorbidities			0.64
Cardiovascular comorbidity	10	7	
DM		3	
No history	9	13	
Etc *	8	17	
Hb (g/dL)	13.47 ± 1.44	12.74 ± 1.87	0.08
WBC (10^3^/uL)	6.13 ± 2.60	6.46 ± 2.63	0.61
PLT (10^3^/uL)	197.66 ± 46.68	242.0 ± 93.11	0.03
Protein (g/dL)	7.02 ± 0.54	7.04 ± 0.77	0.94
Albumin (g/dL)	4.21 ± 0.29	4.08 ± 0.37	0.14
CRP (mg/L)	1.84 ± 4.33	19.10 ± 29.94	0.01
ESR (mm/hr)	13.54 ± 12.35	31.25 ± 28.37	0.01

* Etc: Malignancy, Pulmonary tuberculosis, Ankylosing Spondylitis, Rheumatoid arthritis. BMI; Body Mass Index, PLT; platelet, NSAIDs; Non-steroidal anti-inflammatory drug, DM; diabetes mellitus Hb; hemoglobin, WBC; white blood cells, PLT; platelet, CRP; C-reactive protein, ESR; erythrocyte sedimentation rate.

**Table 2:** Findings of video capsule endoscopy.

Finding of Capsule Endoscopy	*n* = 65
**Negative**	38 (58.6%)
**Mucosal inflammatory lesions**	
Ulcer	10 (15.4%)
Erosions	8 (12.3%)
Ulcer with stricture	4 (6.2%)
Erythema with edema	1 (1.5%)
**Lymphagiectasia**	1 (1.5%)
**Tumorous lesions**	
SET *	2 (3.0%)
Multiple polyps	1 (1.5%)

* SET: subepithelial tumors.

**Table 3:** Univariate analysis of the factors influencing the positive findings of VCE.

Variable	OR	95% CI	*p*
Age	1.017	0.987–1.047	0.28
Gender	0.764	0.282–2.071	0.597
BMI	1.051	0.898–1.230	0.537
Hb	0.752	0.541–1.047	0.091
PLT	1.01	1.001–1.018	0.023
BUN	1.041	0.922–1.175	0.515
Cr	0.154	0.006–3.919	0.257
Protein	1.033	0.468–2.279	0.936
Albumin	0.28	0.054–1.469	0.133
CRP	1.117	1.004–1.243	0.042
ESR	1.045	1.013–1.079	0.006

**Table 4:** Multivariate analysis of the factors influencing the positive findings of VCE.

Variable	OR	95% CI	*p*-Value
Age	0.99	0.95–1.05	0.86
Gender	2.62	0.21–33.27	0.46
BMI	1.02	0.81–1.28	0.89
CRP	1.1	0.95–1.25	0.213
ESR	1.057	1.02–1.1	0.004
PLT	0.99	0.98–1.01	0.37

## Data Availability

The data are not publicly available because of our institutional guidelines.

## Supplementary Materials

The following are available online at https://www.mdpi.com/article/10.3390/diagnostics11112123/s1, Table S1: Characteristics of selected studies, Table S2: Quality of studies by using the QUADAS-2 tool, Table S3: Summary of results from included studies.
