# Supplementary material for: Predictors of Positive Video Capsule Endoscopy Findings for Chronic Unexplained Abdominal Pain: Single-Center Retrospective Study and Meta-Analysis
Source: Diagnostics (Basel). 2021 Nov 16;11(11):2123. doi: 10.3390/diagnostics11112123 (PMC8617728; doi:10.3390/diagnostics11112123)
Supplement: Supplementary file 1 [file diagnostics-11-02123-s001.zip › diagnostics-1427133-supplementary.pdf]

Supplement Table S1. Characteristics of selected studies

Study, year	Design	country	No of centers	Capsule manufacturer	No of patients with CAP	SEX, male/female	Age, mean(range)	Diagnostic yield, No
Shim, 2006	retrospective	korea	Multiple	N/A	110	70/40	50.8 (N/A)	19
Katsinelos, 2011	prospective	greece	Multiple	N/A	72	N/A	45.3 (14-81)	30
Huang, 2018	retrospective	china	Multiple	OMOM	341	47/49	44.54 (18-75)	96

Supplement Table S2. Quality of studies by using the QUADAS-2 tool

study	Risk of BIAS				Applicability concerns		
	Patient selection	Index test	Reference standard	Flow and timing	Patient selection	Index test	Reference standard
Shim, 2006	Low	Low	Low	Low	Low	Low	Low
Katsinelos, 2011	Low	Low	Low	Low	Low	Low	Low
Huang, 2018	Low	Low	Low	Low	Low	Low	Low

Supplement Table S3. Summary of results from included studies

study	Enrolled patients	Overall diagnostic yield	Relevant variables
Shim, K.-N. *et al.*[9]	110	17.3%	weight loss [OR, 18.6 p= 0.02]
Katsinelos, P. *et al.*[10]	72	44.4%	Elevated ESR [OR, 67.9, p<0.001]<br>Elevated CRP [OR, 41.5, p<0.001]
Huang, L. *et al.*[8]	341	28.15%	weight loss [OR,2.827p=0.038]<br>Hypoalbuminemia [OR, 6.142, p=0.008]<br>Elevated ESR [OR,4.025, p=0.016]<br>Elevated CRP [OR, 7.539, p=0.002]
